# Identify contaminants with decontam on the QIIME 2 Framework

**DOI:** 10.1128/mra.01261-25

**Published:** 2026-04-27

**Authors:** Jorden T. Rabasco, Evan Bolyen, J. Gregory Caporaso, Haley Sapers, Benjamin J. Callahan

**Affiliations:** 1 Department of Population Health and Pathobiology, NC State University College of Veterinary Medicine 70727, Raleigh, North Carolina, USA; 2 Pathogen and Microbiome Institute, Northern Arizona University 3356, Flagstaff, Arizona, USA; 3 Department of Biological Sciences, Northern Arizona University 222339, Flagstaff, Arizona, USA; 4 Department of Astronomy and Planetary Science, Northern Arizona University 3356, Flagstaff, Arizona, USA; 5 Bioinformatics Research Center, NC State University 6798 , Raleigh, North Carolina, USA; University of Michigan, Ann Arbor, Michigan, USA

**Keywords:** microbiome, contamination, decontam, QIIME 2, amplicon, metagenome

## Abstract

Here, we present the integration of the decontam method for contaminant identification and a supplemental approach for identifying the source of contaminants in sequencing data within the QIIME 2 Framework for microbiome data science. We demonstrate its use in a tutorial based on the QIIME 2 “Moving Pictures Tutorial” data.

## ANNOUNCEMENT

Contaminant identification, within a sequenced-based experiment, is crucial to an analysis as contaminants can affect scientific interpretations and downstream processes (1–5). While much work has been done in this area (6–8), the use of these techniques has not been consistently implemented. To this end, here, we expanded the contaminant identification options available within the QIIME 2 Framework by incorporating decontam, an established bioinformatic method for identifying contaminants in taxonomic feature tables derived from microbiome sequencing data (either amplicon or metagenome) (7) previously only available as an R package. QIIME 2 is a very widely used platform for microbiome data analysis (9–13). The QIIME 2 Framework (Q2F, currently being rebranded as *rachis*) is a Python-based framework that works on the basis of “plugins” or software packages that ease integration between diverse analysis methods (14). The plugin mechanism has been used to compose prominent bioinformatics software packages from other developers such as DADA2 (15), Deblur (16), and Kraken2 (17). While dedicated contaminant identification methods have not been available within QIIME 2 until recently, with the addition of SCRuB (8), the decontam functionality, is now implemented within the QIIME 2 q2-quality-control plugin (https://github.com/qiime2/q2-quality-control), which is provided by default in both the QIIME 2 and MOSHPIT (18) (i.e., the amplicon and metagenome suites of tools, respectively) distributions. This functionality is, therefore, available for Linux, macOS, and Windows (via WSL) and is available in the Docker container builds provided for all QIIME 2 and MOSHPIT releases. Installation instructions can be found at https://library.qiime2.org/quickstart.

**Fig 1:**
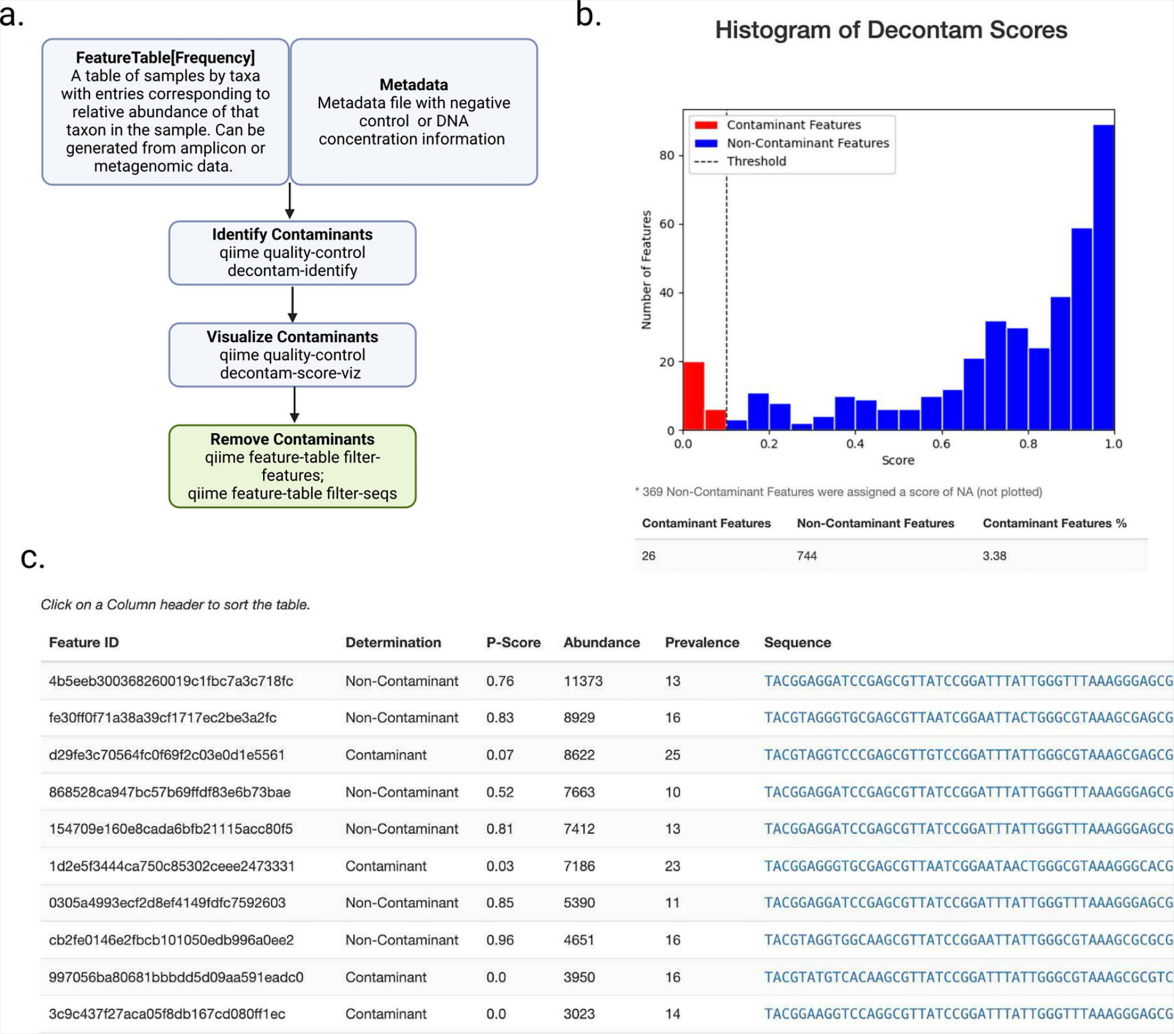
QIIME 2 decontam workflow and example output. (**a**) Standard workflow for using decontam within the QIIME 2 Framework with required inputs. To remove identified contaminants, the actions “qiime feature-table filter-seqs” and “qiime feature-table filter-features” are utilized. (**b**) Histogram of decontam scores produced as output from decontam-score-viz. (**c**) Table of taxonomic features and associated information output by decontam-score-viz.

Three new QIIME 2 actions were implemented—decontam-identify, decontam-score-viz, and decontam-identify-batches. The decontam-identify action, written in Python and R, implements the core decontam functionality of assigning scores to taxonomic features, indicating their consistency with contaminant or non-contaminant origin (7). The decontam-score-viz action—utilizing Python, HTML, CSS, and JavaScript—produces as output a histogram of the decontam scores and a table of taxonomic features along with their decontam scores, abundances, prevalences, and classifications as contaminant or non-contaminant. If representative sequences were provided, the table includes hyperlinks to a web-based BLAST search of those sequences against NCBI’s “core_nt” reference database (Fig. 1b and c). The decontam-identify-batches action is a QIIME 2 pipeline action intended for identification of batch-associated contamination and can aid users in localizing contaminants to specific sections of their measurement protocol such as sequencing run, kit, or date of extraction.

All technical information associated with these actions can be seen in Table 1. To demonstrate the use of the new decontam functionality within QIIME 2, a tutorial was generated using the QIIME 2 Moving Pictures Tutorial data (19) available at https://amplicon-docs.qiime2.org/en/latest/how-to-guides/decontam. This software is made available under the BSD 3-Clause license, and the tutorial is available under the CC-BY license.

**TABLE 1:** Technical information for the actions implemented within the QIIME 2 framework

		Flag	Qiime 2 data type	Description
decontam-identify	Inputs	--i-table	Feature Table [Frequency]	Feature table from which contaminated sequences will be identified from
		--m-metadata-file	METADATA	Metadata file indicating which samples in the experiment are control samples
	Outputs	--o-decontam-scores	FeatureData [DecontamScore]	The resulting table of scores from the decontam algorithm that scores each feature on how likely they are to be a contaminant sequence
decontam-score-viz	Inputs	--i-table	FeatureTable [Frequency]	Feature table from which contaminated sequences will be identified from
		--o-decontam-scores	FeatureData [DecontamScore]	The resulting table of scores from the decontam algorithm that scores each feature on how likely they are to be a contaminant sequence
	Outputs	--o-visualization	VISUALIZATION	Visualization to be rendered via QIIME 2 View
decontam-identify-batches	Inputs	--i-table	Feature Table [Frequency]	Feature table from which contaminated sequences will be identified from
		--m-metadata-file	METADATA	Metadata file indicating which samples in the experiment are control samples
	Outputs	--o-batch-subset-tables	Collection [FeatureTable [Frequency]]	Directory where feature tables split based on metadata and parameter split-column values
		--o-decontam-scores	Collection [FeatureData [DecontamScore]]	The resulting tables of scores from the decontam algorithm that scores each feature on how likely they are to be a contaminant sequence
		--o-score-histograms	VISUALIZATION	Visualization for all data subsets; to be rendered via QIIME 2 View

## Data Availability

Example data sets used for validation and tutorial generation can be found at "https://data.qiime2.org/2024.10/tutorials/moving-pictures/emp-single-end-sequences/sequences.fastq.gz" as part of the QIIME 2 "Moving Pictures Tutorial." These data are a subset of the sequence data and sample metadata that are publicly available under the "Moving Pictures of the Human Microbiome" project [MG-RAST:4457768.3-4459735.3].

## EDITOR'S NOTE

**Editor's Note:** This article was handled by Pat Schloss, who acted as a Guest Editor in consultation with the Editor in Chief, Irene Newton. Dr. Schloss's role as the Guest Editor of this article is outside of his duties as the chair of the Journals Committee.

## References

[1] Weiss S, Amir A, Hyde ER, Metcalf JL, Song SJ, Knight R. 2014. Tracking down the sources of experimental contamination in microbiome studies. Genome Biol 15:564. doi:10.1186/s13059-014-0564-2 25608874 PMC4311479

[2] Salter SJ, Cox MJ, Turek EM, Calus ST, Cookson WO, Moffatt MF, Turner P, Parkhill J, Loman NJ, Walker AW. 2014. Reagent and laboratory contamination can critically impact sequence-based microbiome analyses. BMC Biol 12:87. doi:10.1186/s12915-014-0087-z 25387460 PMC4228153

[3] Karstens L, Asquith M, Davin S, Fair D, Gregory WT, Wolfe AJ, Braun J, McWeeney S. 2019. Controlling for contaminants in low-biomass 16S rRNA gene sequencing experiments. mSystems 4:e00290-19. doi:10.1128/mSystems.00290-19 PMC6550369 31164452

[4] Eisenhofer R, Minich JJ, Marotz C, Cooper A, Knight R, Weyrich LS. 2019. Contamination in low microbial biomass microbiome studies: issues and recommendations. Trends Microbiol 27:105–117. doi:10.1016/j.tim.2018.11.003 30497919

[5] Minich JJ, Sanders JG, Amir A, Humphrey G, Gilbert JA, Knight R. 2019. Quantifying and understanding well-to-well contamination in microbiome research. mSystems 4:e00186-19. doi:10.1128/mSystems.00186-19 PMC6593221 31239396

[6] Minich JJ, Zhu Q, Janssen S, Hendrickson R, Amir A, Vetter R, Hyde J, Doty MM, Stillwell K, Benardini J, Kim JH, Allen EE, Venkateswaran K, Knight R. 2018. KatharoSeq enables high-throughput microbiome analysis from low-biomass samples. mSystems 3:e00218-17. doi:10.1128/mSystems.00218-17 29577086 PMC5864415

[7] Davis NM, Proctor DM, Holmes SP, Relman DA, Callahan BJ. 2018. Simple statistical identification and removal of contaminant sequences in marker-gene and metagenomics data. Microbiome 6:226. doi:10.1186/s40168-018-0605-2 30558668 PMC6298009

[8] Austin GI, Park H, Meydan Y, Seeram D, Sezin T, Lou YC, Firek BA, Morowitz MJ, Banfield JF, Christiano AM, Pe’er I, Uhlemann A-C, Shenhav L, Korem T. 2023. Contamination source modeling with SCRuB improves cancer phenotype prediction from microbiome data. Nat Biotechnol 41:1820–1828. doi:10.1038/s41587-023-01696-w 36928429 PMC10504420

[9] Amos GCA, Logan A, Anwar S, Fritzsche M, Mate R, Bleazard T, Rijpkema S. 2020. Developing standards for the microbiome field. Microbiome 8:98. doi:10.1186/s40168-020-00856-3 32591016 PMC7320585

[10] Eun Y-G, Lee J-W, Kim SW, Hyun D-W, Bae J-W, Lee YC. 2021. Oral microbiome associated with lymph node metastasis in oral squamous cell carcinoma. Sci Rep 11:23176. doi:10.1038/s41598-021-02638-9 34848792 PMC8633319

[11] Bernabeu A, Lledo B, Díaz MC, Lozano FM, Ruiz V, Fuentes A, Lopez-Pineda A, Moliner B, Castillo JC, Ortiz JA, Ten J, Llacer J, Carratala-Munuera C, Orozco-Beltran D, Quesada JA, Bernabeu R. 2019. Effect of the vaginal microbiome on the pregnancy rate in women receiving assisted reproductive treatment. J Assist Reprod Genet 36:2111–2119. doi:10.1007/s10815-019-01564-0 31446545 PMC6823330

[12] Maki KA, Burke LA, Calik MW, Watanabe-Chailland M, Sweeney D, Romick-Rosendale LE, Green SJ, Fink AM. 2020. Sleep fragmentation increases blood pressure and is associated with alterations in the gut microbiome and fecal metabolome in rats. Physiol Genomics 52:280–292. doi:10.1152/physiolgenomics.00039.2020 32567509 PMC7468692

[13] Wu Z, Byrd DA, Wan Y, Ansong D, Clegg‐Lamptey J, Wiafe‐Addai B, Edusei L, Adjei E, Titiloye N, Dedey F, et al.. 2022. The oral microbiome and breast cancer and nonmalignant breast disease, and its relationship with the fecal microbiome in the Ghana Breast Health Study. Intl Journal of Cancer 151:1248–1260. doi:10.1002/ijc.34145 PMC9420782 35657343

[14] Bolyen E, Rideout JR, Dillon MR, Bokulich NA, Abnet CC, Al-Ghalith GA, Alexander H, Alm EJ, Arumugam M, Asnicar F, et al.. 2019. Reproducible, interactive, scalable and extensible microbiome data science using QIIME 2. Nat Biotechnol 37:852–857. doi:10.1038/s41587-019-0209-9 31341288 PMC7015180

[15] Callahan BJ, McMurdie PJ, Rosen MJ, Han AW, Johnson AJA, Holmes SP. 2016. DADA2: High-resolution sample inference from Illumina amplicon data. Nat Methods 13:581–583. doi:10.1038/nmeth.3869 27214047 PMC4927377

[16] Amir A, McDonald D, Navas-Molina JA, Kopylova E, Morton JT, Zech Xu Z, Kightley EP, Thompson LR, Hyde ER, Gonzalez A, Knight R. 2017. Deblur rapidly resolves single-nucleotide community sequence patterns. mSystems 2:e00191–16. doi:10.1128/mSystems.00191-16 28289731 PMC5340863

[17] Wood DE, Lu J, Langmead B. 2019. Improved metagenomic analysis with Kraken 2. Genome Biol 20:257. doi:10.1186/s13059-019-1891-0 31779668 PMC6883579

[18] Ziemski M, Gehret L, Simard A, Dau SC, Risch V, Grabocka D, Matzoros C, Wood C, Cabrera PM, Hernández-Velázquez R, Herman C, Evans K, Robeson MS 2nd, Bolyen E, Caporaso JG, Bokulich NA. 2025. MOSHPIT: accessible, reproducible metagenome data science on the QIIME 2 framework. bioRxiv:2025.01.27.635007. doi:10.1101/2025.01.27.63500742785295

[19] Caporaso JG, Lauber CL, Costello EK, Berg-Lyons D, Gonzalez A, Stombaugh J, Knights D, Gajer P, Ravel J, Fierer N, Gordon JI, Knight R. 2011. Moving pictures of the human microbiome. Genome Biol 12:R50. doi:10.1186/gb-2011-12-5-r50 21624126 PMC3271711

